# Emotion Regulation Difficulties in Boys with Oppositional Defiant Disorder/Conduct Disorder and the Relation with Comorbid Autism Traits and Attention Deficit Traits

**DOI:** 10.1371/journal.pone.0159323

**Published:** 2016-07-15

**Authors:** Jantiene Schoorl, Sophie van Rijn, Minet de Wied, Stephanie van Goozen, Hanna Swaab

**Affiliations:** 1 Department of Clinical Child and Adolescent Studies, Leiden University, Leiden, The Netherlands; 2 Leiden Institute for Brain and Cognition, Leiden University, Leiden, The Netherlands; 3 Department of Adolescent Development, Utrecht University, Utrecht, The Netherlands; 4 School of Psychology, Cardiff University, Cardiff, United Kingdom; Bellvitge Biomedical Research Institute-IDIBELL, SPAIN

## Abstract

Previous research has pointed towards a link between emotion dysregulation and aggressive behavior in children. Emotion regulation difficulties are not specific for children with persistent aggression problems, i.e. oppositional defiant disorder or conduct disorder (ODD/CD), children with other psychiatric conditions, such as autism spectrum disorders or attention-deficit/hyperactivity disorder, have emotion regulation difficulties too. On a behavioral level some overlap exists between these disorders and comorbidity is high. The aim of this study was therefore twofold: 1) to examine emotion regulation difficulties in 65 boys with ODD/CD in comparison to a non-clinical control group (NC) of 38 boys (8–12 years) using a performance measure (Ultimatum Game), parent report and self-report, and 2) to establish to what extent emotion regulation in the ODD/CD group was correlated with severity of autism and/or attention deficit traits. Results on the Ultimatum Game showed that the ODD/CD group rejected more ambiguous offers than the NC group, which is seen as an indication of poor emotion regulation. Parents also reported that the ODD/CD group experienced more emotion regulation problems in daily life than the NC group. In contrast to these cognitive and behavioral measures, self-reports did not reveal any difference, indicating that boys with ODD/CD do not perceive themselves as having impairments in regulating their emotions. Emotional decision making within the ODD/CD group was not related to variation in autism or attention deficit traits. These results support the idea that emotion dysregulation is an important problem within ODD/CD, yet boys with ODD/CD have reduced awareness of this.

## Introduction

Children with Oppositional Defiant Disorder (ODD) and Conduct Disorder (CD) are characterized by persistent antisocial and aggressive behaviors [1]. Children suffering from ODD and CD are at risk for numerous negative outcomes, such as delinquency, unemployment, depression, anxiety and other psychiatric problems [2]. Identifying risk factors for antisocial and aggressive behavior that can be targets for potential change is therefore important. Recently, it has been proposed that problems in emotion regulation, referring to “the processes by which individuals influence which emotions they have, when they have them, and how they experience and express these emotions” [3], may be an important mechanism driving behavioral problems in ODD [4–7]. Impaired emotion regulation skills are also thought to drive behavior problems in children with other psychiatric disorders, such as autism spectrum disorders (ASD) [8] and attention-deficit/hyperactivity disorder (ADHD) [9]. Therefore, there is a need to investigate if emotion regulation difficulties exist in those with ODD/CD, and to what extent emotion regulation difficulties are related to comorbid autism and attention deficit traits in those with ODD/CD, using cognitive, behavioral and self-report measures of emotion regulation.

Emotion regulation is necessary for psychological well-being and social functioning [10]. Although emotion regulation strategies can be used deliberately, often these processes operate unconsciously. In everyday life we are regularly confronted with situations eliciting emotions. Emotion regulation helps us to respond to those emotions in a socially acceptable and flexible way [3]. Impaired emotion regulation skills in children have been associated with reduced prosocial behavior [11], academic success [12], social competence [13], quality of social relationships [14], and increased vulnerability for psychopathology [15].

The review of Roll, Koglin [16] showed that there are several empirical studies in support of the hypothesis that emotion regulation may be compromised in children with aggressive and antisocial behavior. Effective emotion regulation starts with emotional awareness, defined as attention to and insight in one’s own emotional responses and functioning [17]. Insufficient emotional awareness may lead to handling unpleasant emotions with impulsive acting-out behavior, because of misinterpreted internal and external emotional cues [18]. Factor, Rosen [19] indeed found that self-reported impaired emotional awareness was associated with comorbid externalizing disorders in a child population with ADHD. Male adolescent offenders reported substantially more problems in identifying feelings as compared to a non-clinical control group [20]. Manninen, Therman [18] found that male adolescents with severe behavior problems had more problems in describing feelings to others than controls.

Emotional awareness influences the processes of emotion regulation; after becoming aware of emotions, one needs to label them correctly and decide which responses or behaviors are necessary/appropriate or need to be inhibited [3]. Children with aggression problems have been found to be less likely to inhibit emotional reactivity and used less effective or more inappropriate regulatory strategies [11, 21]. Furthermore, McLaughlin, Hatzenbuehler [22] concluded that emotion dysregulation is a strong predictor of aggressive behavior in an adolescent community sample and not vice versa.

Emotion dysregulation is not specific for children with ODD/CD. Children with other psychiatric disorders, such as ADHD or ASD, also have emotion regulation difficulties [1, 9, 23]. Comorbidity rates of ODD or CD in children with ADHD is high (59% and 43% respectively) [9]. Aggression is displayed in over 50% of the children with ASD [24] and according to a review containing seven studies one in four meets ODD or CD criteria [25]. Another study even reported that 41% of the children with ASD displayed clinical levels of symptoms of ODD/CD [26]. According to Barkley [9] children with ADHD lack the capacity for inhibition, making it difficult for them to delay a response long enough to gather information necessary for understanding emotionally charged situations. Children with ASD are prone to react impulsively to emotional stimuli with tantrums, aggression or self-injury, which is thought to result from impaired emotion regulation skills [8]. Recently, studies have suggested that ADHD is associated with emotional dysregulation only in the presence of a comorbid disorder, such as ODD, anxiety or depression [27] or CD [28].

Based on these findings, the question arises whether emotion regulation deficits in children with ODD/CD are characteristic of the ODD/CD group as a whole, or whether these deficits are only present in subgroups, for example those with autism spectrum traits or attention deficit traits [29]. Therefore, by studying emotion regulation in children with ODD/CD one needs to include autism traits and attention deficit traits as well.

Studies on emotional awareness and emotion regulation, as reviewed above, have primarily used (self-report) questionnaires, with very few assessing the ability to process and regulate emotions. Therefore, in this study, emotion regulation was examined from three perspectives using a performance measure, a parent report and self-reports of emotion regulation. First, we used an emotional decision making task: the Ultimatum Game (UG), in which subjects need to decide if they accept or reject fair, ambiguous or unfair money offers [30]. A number of studies provided evidence that emotion regulation processes are a critical component of the UG; the percentage of accepted unfair offers can be influenced by focusing on specific emotion regulation strategies (reappraisal instead of suppression) [31, 32], rejection of unfair offers increased when feelings of sadness are induced [33], neural substrates known to be associated with negative emotions were activated when unfair offers are presented [30] and lesions in these areas resulted in higher rejection rates [34]. Therefore the UG is a useful instrument in studying emotion regulation [28, 34–36] and provides insight into emotional reactivity that results from automatic regulation processes that can take place without monitoring, insight or awareness [37]. Secondly, we used a parent report measure of children’s emotion regulation in daily life. Third, we used a child self-report measure of emotion regulation and emotional awareness, providing insight in self-perceived emotion regulation skills. These three types of measures will help us in disentangling emotion regulation difficulties in children with ODD/CD. It is hypothesized that children with ODD/CD have emotion regulation difficulties, as evident in the parental report, self-reports and emotional decision making task. Furthermore, autism traits and attention deficits traits were related to the three emotion regulation measures within those with ODD/CD to explore if emotion regulation difficulties are related to ODD/CD or if they underlie other behavioral problems, such as attention deficit traits and autism traits.

## Method

The current study was approved by the Medical Ethical Committee of Leiden University Medical Centre (LUMC). Parents and boys who were twelve years old signed an informed consent according to the Declaration of Helsinki prior to participation. Participation was voluntary.

### Participants

#### ODD/CD group

Inclusion criteria for the oppositional defiant disorder and conduct disorder (ODD/CD) group was a diagnosis of ODD or CD on the Diagnostic Interview Schedule for Children (DISC-IV) [38], estimated IQ >70 and aged between 8 and 12 years old; This resulted in a total of 65 included boys with ODD/CD. All boys with ODD/CD met criteria for ODD, 22 (34%) also met criteria for CD. Other comorbid disorders were: ADHD (*n =* 45, 69%), anxiety (*n =* 38, 58%), depression (*n =* 9, 14%), autism traits (mild: *n =* 22, 34%, severe: *n =* 20, 31%), and other disorders such as eating and tic disorders (*n =* 18, 28%). Twenty-four boys (37%) used psychostimulants and four (6%) used atypical antipsychotics.

#### Non-clinical control group

Inclusion criteria for the non-clinical control (NC) group were IQ >70, aged between 8 and 12 years old, and they were screened to have no aggression expressed as a diagnosis of ODD or CD, a score outside the normal range (T>60) on the externalizing scale of the Child Behavior Checklist (CBCL/6-18) or Teacher Report Form (TRF/6-18) [39] and low levels of autism traits expressed as a score in the normal range (T<60) on the Social Responsiveness Scale (SRS) [40]; This resulted in a NC group of 38 boys. Two boys (5%) in the NC group used psychostimulants.

The ODD/CD group was similar in age (*M* = 10.3, *SD* = 1.28) and percentage of Caucasians (62%) compared to the NC Group (age *M* = 10.1, *SD* = 1.27), *t* = .79, *p =* .434; (Caucasian 68%), *χ* = .49, *p* = .482. The ODD/CD group did have lower estimated IQ scores (*M* = 95.6, *SD* = 14.23) than the NC group (*M* = 104.1, *SD* = 12.07), *t* = -3.08, *p* = .003.

### Recruitment and procedures

Boys with ODD/CD were recruited at clinical health centers (*n* = 22), special education schools (*n* = 31) and regular elementary schools (*n* = 12). NC’s were solely recruited at regular elementary schools (*n* = 38). Boys referred through clinical centers were initially screened using the CBCL [39], only those who scored above borderline cut off point on the externalizing scale were also subjected to the DISC interview [38]. Those who fulfilled ODD or CD criteria were asked to participate in this study.

All participating boys were invited to visit Leiden University for one day with one of their parents. During this day parents filled out questionnaires and completed the DISC-IV interview. The boys completed computer tasks and filled out questionnaires. Within two weeks the second session took place either at the child’s school or at the clinical health center. The teacher of the child filled out the TRF [39].

### Measures

*IQ* was measured with Vocabulary and Block Design, two subtests of the Wechsler Intelligence Scale for Children (WISC-IV) [41]. These subtests have been found to provide a good estimation of full scale IQ scores [42].

*Autism traits* were measured by the raw scores of the SRS, a parent questionnaire for assessing autistic traits [40]. The SRS has good validity [43], and has good reliability [40]. The coefficient alpha (α) for the whole sample was.83.

*Attention deficit traits* were measured using raw scores of the subscale ‘attention problems’ (α = .94) of the TRF/6-18 [39].

*Emotional decision making* was measured by a child version of the Ultimatum Game (UG) [30]. In this computer game a fictitious proposer (21 times a computer and 21 times a child) offers to split 10 euro. The offer was 14 times fair (5–5) 14 times ambiguous (7–3) and 14 times extremely unfair (9–1). The task was programmed in E-prime 2.0 and was administered on a laptop. Offers and proposers were randomly assigned. If the boy accepted the offer the money was split as proposed, if the boy rejected the offer no one received money. Boys were instructed to earn as much money as they could and should therefore accept any offer made. However, previous studies have shown that fifty percent of the time low offers are rejected (emotional decision) instead of accepted (cognitive decision), thereby providing a measure of cognition-emotion interactions in strategic decision making. Parameters were percentages of accepted fair, ambiguous and unfair offers (0–100%).

*Parental report of emotion regulation in daily life* was measured by the subscale Emotional Control of the Behavior Rating Inventory of Executive Function (BRIEF) [44]. Parents rated their child’s behavior on a three-point scale (never, sometimes, often) on 10 questions about emotion regulation. Raw scores were used. Mean internal consistency ratings, three week test-retest correlations, convergent and discriminant validity of this scale are good [44]. Coefficient alpha was .94 for the whole sample.

#### Children’s self-reported emotion regulation

*Cognitive emotion regulation* was measured by the Cognitive Emotion Regulation Questionnaire children’s version (CERQ-k) [45], a self-report containing 36 items covering nine cognitive emotion regulation strategies: self-blame, acceptance, rumination, positive refocusing, refocus on planning, positive reappraisal, putting into perspective, catastrophizing and blaming others. Children answered what they would think after experiencing negative incidents/situations by using a five-point Likert scale ranging from almost never (1) till almost always true (5). The different strategies all have good internal consistencies [45]. Coefficient alphas of the subscales varied between .63 and .74 for the whole sample.

*Emotional awareness* was measured by the Emotional Awareness Questionnaire (EAQ) [46]. Children had to rate if all 30 items were true about them on a three-point scale (never, sometimes and often), resulting in six aspects of emotional functioning: Differentiating Emotions (α = .99), Not Hiding Emotions (α = .99), Analyses of Emotions (α = .76), Attention to Others' Emotions (α = .68), Verbal Sharing of Emotions (α = .59) and Bodily Awareness of Emotions (α = .65). The EAQ showed good psychometric properties, good criterion validity and good concurrent validity [46].

### Statistical analysis

All data followed normal distributions, except for fair (positively skewed) and unfair offers (negatively skewed) in emotional decision making. First, group comparisons between ODD/CD and NC group were calculated. Kruskal Wallis H tests (nonparametric) did not reveal different results for fair and unfair offers than parametric t-tests, therefore, a repeated measures ANOVA was performed to compare ODD/CD and NC group on their decision making under influence of emotions. Then, differences in emotion regulation in daily life reported by parents were calculated with an ANCOVA. Next, a MANCOVA was performed to test whether ODD/CD and NC group differed from each other in their emotional awareness and self-reported cognitive emotion regulation. Finally, correlations were calculated within the ODD/CD group between the emotion regulation measures on which the ODD/CD and NC group significantly differed, and autism traits and attention deficit traits.

## Results

A multivariate analysis revealed that boys with ODD/CD who used medication did not differ on any of the emotion regulation measures from those who did not use medication, *F* = .74, *p* = .717, nor did the results differ when the two NC boys who used medication were included or excluded in the group comparisons of ODD/CD versus NC. Therefore, medication was not controlled for in subsequent analyses. To verify that the place of recruitment was not associated with any of the outcome variables, a multivariate analysis was conducted with all emotion regulation measures (emotional decision making, parent reported emotion regulation in daily life and self-reports) and place of recruitment as the dependent variable. Place of recruitment was not related to emotion regulation, *F* = .95, *p* = .559. Because IQ was significantly higher in the control group, a correlation was first calculated to check if IQ was correlated with any of the dependent variables. IQ significantly correlated with emotion regulation in daily life rated by parents, *r* = -.22, *p* = .028, and the subscale ‘Analysis of Emotions’ of the EAQ, *r* = .43, *p* < .001, therefore IQ was controlled for in the analyses with these variables.

### Emotional decision making

A multivariate test revealed that for fair, ambiguous and unfair offers percentages of accepted offers were not different for pc and human proposers, *F* (3, 100) = 2.17, *p* = .096. Therefore, pc and human offers were collapsed.

**Fig 1:**
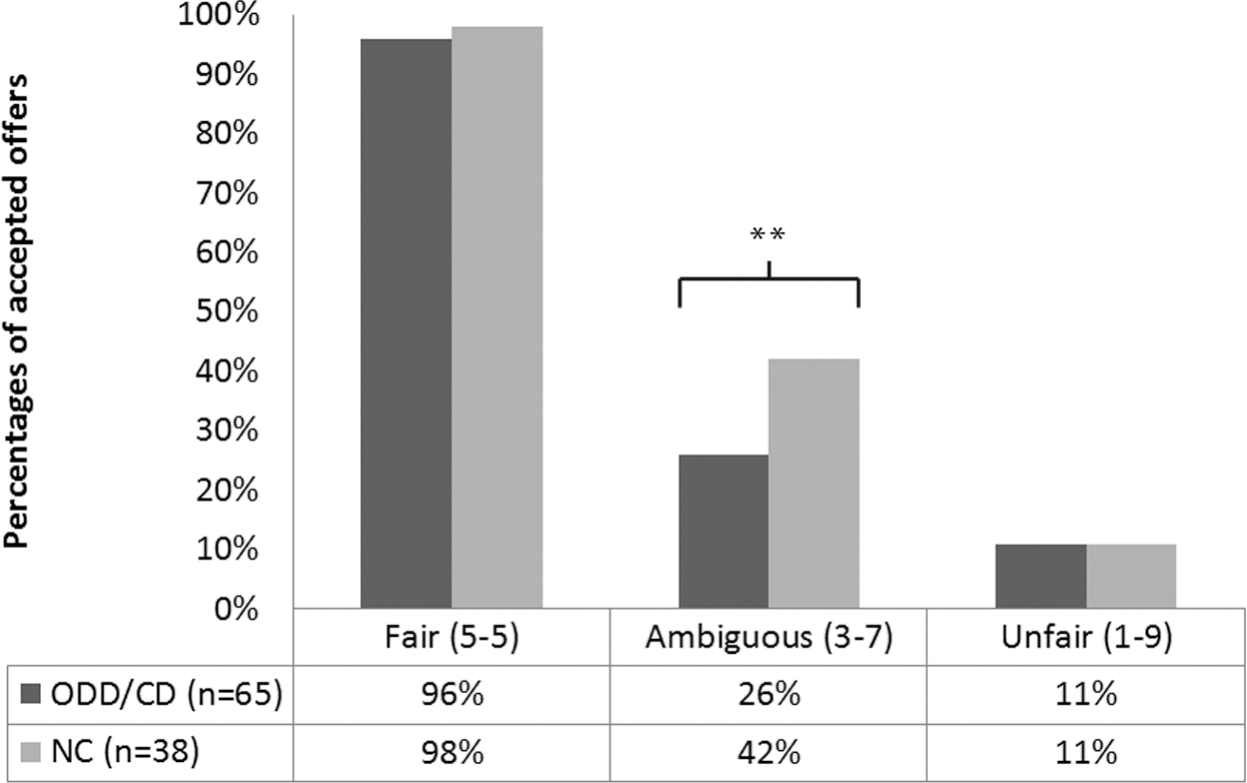
Percentages of accepted offers in the ODD/CD and NC group (Ultimatum Game). ** p < .01.

A repeated measures ANOVA showed a significant main effect of condition (fair, ambiguous, unfair) *F*(1.77, 179.05) = 598.24, *p* < .001, with a large effect, *η*^*2*^ = .86, group (ODD/CD vs NC) *F*(1, 101) = 3.91, *p* = .051, *η*^*2*^ = .04 and condition by group *F*(1.77, 179.05) = 5.59, *p* = .004, *η*^*2*^ = .05. Post hoc t-tests showed that only in ambiguous situations the ODD/CD group (*M* = 26.2, *SD* = 26.00) rejected more offers than NC group (*M* = 42.3, *SD* = 29.15), *t* = -2.96, *p* = .004 (see Fig 1).

### Parental report of emotion regulation in daily life

Results revealed that there was a significant main effect of group *F*(1, 100) = 79.97, *p* < .001, *η*^*2*^ = .44, but not for IQ *F*(1, 100) = .05, *p* = .823. Parents of boys with ODD/CD (*M* = 21.3, *SD* = 5.41) reported significantly more emotion regulation problems than parents of controls (*M* = 12.4, *SD* = 2.63).

### Self-reported emotion regulation skills

Three boys with ODD/CD did not fill out the questionnaires. The MANCOVA revealed that there was a significant effect of IQ *F*(15,83) = 2.26, *p* = .010, but no effect of group *F*(15, 83) = .70 *p* = .696 (Table 1). In other words, boys with ODD/CD did not report significant impairments in emotion regulation strategies or emotional awareness as compared to controls.

**Table 1:** Emotional awareness and cognitive emotion regulation in the ODD/CD (n = 61) and NC group (n = 38).

		DBD	NC	*F*	*p*
EAQ	Differentiating Emotions	1.8 ± .41	1.6 ± .46	2.70	.104
	Not Hiding Emotions	2.1 ± .45	1.9 ± .46	3.00	.087
	Analyses of Emotions	2.1 ± .54	2.3 ± .44	.21	.648
	Attending to Others' Emotion	1.9 ± .34	1.9 ± .31	.99	.323
	Verbal Sharing of Emotions	1.9 ± .50	1.9 ± .40	.01	.915
	Bodily Awareness of Emotions	1.9 ± .44	1.8 ± .40	2.62	.109
CERQ	Self-blame	9.8 ± 3.48	9.1 ± 3.34	.76	.386
	Acceptance	11.4 ± 4.16	10.9 ± 3.76	.34	.563
	Rumination	11.6 ± 4.07	11.5 ± 3.64	.05	.820
	Positive refocusing	13.9 ± 3.69	14.0 ± 4.13	.02	.900
	Refocus on planning	13.03 ± 4.30	13.4 ± 3.60	.01	.926
	Positive reappraisal	11.1 ± 4.38	11.6 ± 4.00	.11	.740
	Putting into perspective	12.2 ± 4.23	11.6 ± 4.00	.38	.539
	Catastrophizing	10.9 ± 4.47	9.5 ± 3.64	1.96	.165
	Other blame	8.4 ± 3.58	7.9 ± 2.94	.61	.437

EAQ, Emotional Awareness Questionnaire; CERQ, Cognitive Emotion Regulation Questionnaire.

### Emotion regulation in relation to autism traits and attention deficit traits

Finally, we examined the possibility that the group differences in emotional decision making in ambiguous situations and parent reported emotion regulation problems in daily life were related to autism traits or attention deficit traits. Therefore, a correlation analysis was performed within the ODD/CD group to examine the relation between the two emotion regulation variables and autism traits and attention deficit traits. Emotional decision making turned out to be not related to autism traits, *r* = .02, *p* = .862 or attention deficit traits, *r* = .09, *p* = .504. Parent reported emotion regulation in daily life was related to (parent reported) autism traits, *r* = .44, *p* < .001, but not to (teacher reported) attention deficit traits, *r* = -.04, *p* = .802.

## Discussion

Previous research has suggested a link between emotion dysregulation and aggressive behavior in children, though these studies mainly focused on parent and self-reports of emotion regulation (e.g. [16, 47]. Therefore, our first aim was to assess emotion regulation using three approaches, including not only a parent report about emotion regulation in daily life and self-reported emotion regulation skills, but also a performance based emotional decision making task. Because emotion regulation difficulties may not be specific for boys with ODD/CD, but may also characterize children with other psychiatric conditions, such as ASD or ADHD, the second aim of the current study was to examine to what extent their emotion regulation difficulties were affected by co-occurring autism traits and attention deficit traits.

The main findings of this study are that boys with ODD/CD showed impaired emotional decision making and emotion regulation in daily life, but have reduced awareness of this since they did not report impairments themselves. Furthermore, autism and attention deficit traits were not related to emotional decision making in boys with ODD/CD.

First, group comparisons revealed that boys with ODD/CD had more problems in emotional decision making compared to controls; Boys with ODD/CD rejected more money offers of the ambiguous proposals than controls. Fair and unfair proposals did not differ between both groups, indicating that boys with ODD/CD do not reject more offers in general. FMRI research shows that the area’s activated in the brain when unfair offers are proposed are also activated when negative emotions are experienced [30, 34], indicating that emotion regulation difficulties may lie at the basis of this difference. Extremely unfair conditions resulted in emotion regulation failure in both groups, which has often been found in studies using the ultimatum game [30, 31, 48]. However, when the situation was ambiguous boys with ODD/CD performed worse than controls. Everyday life situations that require emotion regulation are often ambiguous too, and may therefore be most prone to deficiencies in emotion regulation. Problems in regulating emotional state might help explain ODD/CD symptoms. ODD symptoms for example include losing temper, irritability, easily annoyed [1], symptoms that are closely linked to emotion dysregulation [9]. The antisocial and aggressive behavior of boys with ODD/CD may be in part caused by deficiencies in any of the steps that are part of emotion regulation, i.e. misinterpreted internal and external emotional cues [18], limited range of strategies [11, 21] and lack of behavioral control [11], leading to handling unpleasant emotions with impulsive acting-out behavior. Boys with ODD/CD have also been reported to have difficulties in the processing of social information [49]. For example, some boys with ODD/CD have a hostile attribution bias [50], which may influence their appraisal of ambiguous situations [51].

In line with our hypothesis of deficiencies in regulating emotional state, parents of boys with ODD/CD reported more emotion regulation problems in daily life than controls. Interestingly, when focusing on self-report measures, boys with ODD/CD scored similar to controls on self-perceived emotional awareness and the use of cognitive emotion regulation strategies. This is interesting, because it indicates that boys with ODD/CD, who have deficient emotion regulation, may in addition have difficulties in reflecting on emotion regulation. Regulation requires self-reflection, that is the knowledge about your own feelings, desires and impulses [52]. Since these abilities are core to emotion regulation [3] this might be difficult when emotion regulation is impaired. So the null finding in self-reported emotion regulation skills might indicate that boys with ODD/CD are not able to reflect upon their own emotion regulation properly, which in combination with the worse performance on the emotional decision making task and worse emotion regulating in daily life, supports the idea of pervasive emotion dysregulation.

Our findings of impaired emotion regulation in boys with ODD/CD is in line with the review of Roll, Koglin (16) and other studies showing that (childhood) aggression is associated with less effective or more inappropriate regulatory strategies [11, 21, 22] and reduced emotional awareness [19, 20, 22], although Zeman, Shipman [53] did not find emotional awareness to be related to externalizing problem behavior. In some what older children, male adolescent (offender and severe ODD/CD) populations, an association between aggression and self-reported emotion dysregulation was found [18, 20]. So, we cannot exclude that self-reflection might improve with age.

Second, we examined whether autism traits and attention deficit traits were related to emotion regulation difficulties in the ODD/CD group. It appeared that emotional decision making was not related to autism traits and attention deficit traits in boys with ODD/CD. Parent reported emotion regulation in daily life was also not related to attention deficit traits, but there was a relation with autism traits. Although this result is in line with the expectation that children with autism have impaired emotion regulation skills [8], we cannot rule out that this finding might be caused by an informant bias; autism traits were obtained by parents as well. Therefore, we think the objective measure of emotion regulation was more informative. Nevertheless, attention deficit traits was not related to emotional decision making and parent reported emotion regulation in daily life in boys with ODD/CD. This is in line with other studies suggesting that ADHD is associated with emotion dysregulation only when a comorbid disorder, like ODD [24] or CD [28], is present.

A strength of the current study is that we investigated emotion regulation at three perspectives: objective emotional decision making, parental reported emotion regulation in daily life and self-perceived emotion regulating skills. Parent and child measures revealed different results in the current study. It has often been found that parent- and child-reports do not correlate highly [54]. Although there is no golden standard to guide what informant or measure is best to be used, objective measures, like the emotional decision making task in this study, are free from biases found in questionnaires and are therefore a useful addition. Thought we think that emotions influenced the decision making in the Ultimatum Game [30, 34], we did not measure their actual emotional state under the three conditions, so we cannot be sure mood changed and influenced their decision making. Also, the amount of rejected offers has been used as an indication of aggression in some studies (e.g. [55]). If we would redefine rejection as aggression we would conclude that boys with ODD/CD are only more aggressive than controls in ambiguous situations, in extremely unfair situations both groups did not differ. However, in real life they would differ also, or most certainly, in these situations. In the current study we used a self-report questionnaire that has been validated only in normal child populations (CERQ). Even though one may argue that self-reflection may have not fully developed at this age (8–12 years old), studies have successfully used self-report questionnaires about emotional awareness (EAQ) or expressiveness in psychiatric child populations of similar age: autism [56] and ADHD [19]. Although children with autism display similar behaviors like children with ODD/CD, e.g. oppositional, noncompliant, aggressive behaviors, the function of these behaviors might differ [25]. It is important to make sure that these behaviors occur outside of restricted and repetitive behaviors and interests that are inherent to autism. In the current study we did not control for these functions of antisocial and aggressive behavior. Future research should take into account whether the aggressive behavior (also) occurs outside the restricted and repetitive behaviors and interests when studying children with ODD/CD and autism.

The findings of the current study support the idea that emotion dysregulation is an important problem in those with ODD/CD. Both the cognitive emotion regulation task and parental reported emotion regulation pointed towards impairments in emotion regulation, yet boys with ODD/CD have reduced awareness of this. This is may be important information for diagnostics and treatment. Boys with ODD/CD may have difficulty reflecting upon their emotion regulation skills adequately. In treatment facilities it might therefore be better to use other informants than the child itself. Also, treatment programs designed for improving emotion regulation should not be limited to learn appropriate emotion regulation skills but also include becoming aware of emotions and the coping behavior being used. Improving emotion regulation is important because this may help reducing aggressive and antisocial behavior if children learn how to respond to emotions in everyday life in a socially acceptable and flexible way.
